# Optical Properties of Porous Alumina Assisted Niobia Nanostructured Films–Designing 2-D Photonic Crystals Based on Hexagonally Arranged Nanocolumns

**DOI:** 10.3390/mi12060589

**Published:** 2021-05-21

**Authors:** Andrei Pligovka, Alexander Poznyak, Małgorzata Norek

**Affiliations:** 1 Research and Development Laboratory 4.10 “Nanotechnologies”, Belarusian State University of Informatics and Radioelectronics, 6 Brovki Str., 220013 Minsk, Belarus; poznyak@bsuir.by; 2 Department of Micro- and Nanoelectronics, Belarusian State University of Informatics and Radioelectronics, 6 Brovki Str., 220013 Minsk, Belarus; 3 Department of Electronic Technology and Engineering, Belarusian State University of Informatics and Radioelectronics, 6 Brovki Str., 220013 Minsk, Belarus; 4 Institute of Materials Science and Engineering, Faculty of Advanced Technologies and Chemistry, Military University of Technology, 2 Kaliskiego Str., 00-908 Warsaw, Poland; malgorzata.norek@wat.edu.pl

**Keywords:** aluminum oxide, niobium oxide, refractive index, band gap, oxalic acid, nanowires, niobium, NbO_2_, niobium dioxide, anodization

## Abstract

Three types of niobia nanostructured films (so-called native, planarized, and column-like) were formed on glass substrates by porous alumina assisted anodizing in a 0.2 M aqueous solution of oxalic acid in a potentiostatic mode at a 53 V and then reanodizing in an electrolyte containing 0.5 M boric acid and 0.05 M sodium tetraborate in a potentiodynamic mode by raising the voltage to 230 V, and chemical post-processing. Anodic behaviors, morphology, and optical properties of the films have been investigated. The interference pattern of native film served as the basis for calculating the effective refractive index which varies within 1.75–1.54 in the wavelength range 190–1100 nm. Refractive index spectral characteristics made it possible to distinguish a number of absorbance bands of the native film. Based on the analysis of literature data, the identified oxide absorbance bands were assigned. The effective refractive index of native film was also calculated using the effective-medium models, and was in the range of 1.63–1.68. The reflectance spectra of all films show peaks in short- and long-wave regions. The presence of these peaks is due to the periodically varying refractive index in the layers of films in two dimensions. FDTD simulation was carried out and the morphology of a potential 2-D photonic crystal with 92% (wavelength 462 nm) reflectance, based on the third type of films, was proposed.

## 1. Introduction

Alumina is transparent in the visible spectrum region, thermally stable material, characterized by high hardness and low chemical reactivity [1,2,3,4]. Alumina is applied in the production of ceramics, sitalls, glasses [4], refractory materials [5,6,7], abrasive materials [2], adsorbents [2,4,8], including chromatography [2,9], catalyst carriers and catalytic active [2,4,10], and filter materials [6]. Niobium entities, including oxides, are distinguished by even higher chemical stability [1], and are promising for application in heterogeneous catalysis [11,12,13]. One side band-gap energy of niobia is close of 3.4 eV [8], a value close to that of TiO_2_ (about 3.2 eV [14]) and is, therefore, suitable for use as a photocatalyst under UV light [15,16,17]. Therefore, pentavalent niobia is considered as a substitute for titanium dioxide in many applications [18]. The optical, structural, and electrochromic properties of the different crystalline polymorphic forms of the niobia films make them attractive for optical applications [19,20]. There are various methods for the oxide materials synthesis, in particular, alumina and niobia: the metal thermal oxidation with oxygen to obtain an oxide in the highest oxidation state [18,21] and metal subsequent sintering (sinter) with higher oxide in vacuum in required proportion to obtain oxides in an intermediate oxidation state [20], spray pyrolysis deposition [22,23], atomic-layer deposition [24,25], and plasma-enhanced atomic layer deposition [26], magnetron sputtering technique [18,27,28,29], and reactive magnetron sputter process [30], chemical precipitation from solution [18,31], the controlled precipitation method [16], sol-gel method [4,10,18,32,33], polymeric precursors (Pechini) method [32], hydro-thermal (in water) and solvothermal (in other solvents such as acetone and isopropyl alcohol) synthesis techniques [18], extraction from natural minerals [4], chemical vapor deposition [19,34], and plasma-enhanced chemical vapor deposition [35], pulsed laser deposition [36,37,38], electron beam evaporation [22], molecular beam epitaxy [39], electrodeposition [18]. 

In this work, an electrochemical method for oxide materials formation is of interest. One of the most common methods for synthesizing oxide materials is anodizing [40] due to easy implementation and cost effectiveness. Anodic oxides can be continuous, porous, or tubular, depending on the conditions of the anodizing process. Features of electrochemical behavior of valve metals (Al, Nb, Ta, Ti, etc.), micro and nanodispersed, nanostructured oxides, including porous and tubular anodic oxides and composite materials based on them, are the objects of numerous studies [41,42,43,44,45,46,47,48,49,50,51] and review publications [11,12,18,52,53,54,55,56,57,58]. Particular interest in such materials is due to the fact that they have a quasi-regular structure, which is formed in the process of production as a result of self-organization processes [59]. This spontaneously emerging structure is controllable in various ways within wide limits [33,60,61], which allows the synthesis of composite and functional materials [43] for electronic devices [62,63], various kinds of sensors [54,64,65,66,67,68], and other optically active materials [29,69,70,71,72,73,74,75,76,77], including metamaterials [78] and materials for solar cells and solar fuel production [53,54,79] and other energy storage applications [55,80,81]. It should be noted that the porous structure of native X-ray amorphous porous-alumina (PA) makes it an anisotropic material [82], imparting properties characteristic of anisotropic crystals, including, for example, birefringence [83,84]. Among the objects of nano-optics, photonic crystals (PCs) that have a spatially periodic refractive index, with intervals on the order of the wavelength of light, possessing a photonic band gap [85,86], are of interest. This research area began relatively recently in 1987 with two publications, issued one after another with a small interval and independently of each other [87,88], and is intensively developing at the present time. Both active theoretical studies and properties modeling of PCs [89,90,91] and experimental studies of their spectral characteristics are underway. The combination of various dielectrics with air or other dielectric material is used to create PCs, for example, metal oxides [69,76], silicon and SiO_2_ [92,93,94], SiC [95], polymer [96], GaAs [97], nanochannel glass [98], single crystal diamond [99], Ge/Si self-assembled islands grown by chemical vapor deposition with the air filling factor between 23% and 81% [100], a triangular array of air cylinders etched through laser-like Ga(Al)As waveguiding heterostructure [101]. The possibility of developing metal-dielectric PCs [87], as well as temperature- and magnetic-field-controlled PCs based on high-temperature superconductors [102], is considered. Improvements in organic light-emitting diodes efficiency are studied via the introduction of PC layers [103]. There is a report on the construction of a 2-D PC diode laser [104].

It can be noted that PA and niobia are highly promising for use by lasing, in nanooptic and nanophotonic PCs [3,54,58,67,73,74,76,105,106,107,108,109]. On the other hand, PA, the optical and other properties of which have been well studied today, can become a suitable assistant (template) for obtaining nanostructures [52,95,110,111], including those based on anodic niobia or/and nanocomposites based on a combination of both of these anodic oxides. At the same time, PA, acting as an assistant in the process of creating a nanostructure from anodic niobia or a similar valve metal, can either play an auxiliary role by only setting the morphological parameters of the formed nanostructures, or also act as an addition to niobia, which improves certain properties. For example, a similar idea was implemented in ref. [105].

In this study, PA assisted niobia nanostructured films of three types were fabricated by a two-step anodizing. Next, morphology and optical characteristics in the UV-near-IR range of the films were investigated with the aim of further possible formation of 2-D PCs.

## 2. Materials and Methods

### 2.1. Film Preparation

**Figure 1:**
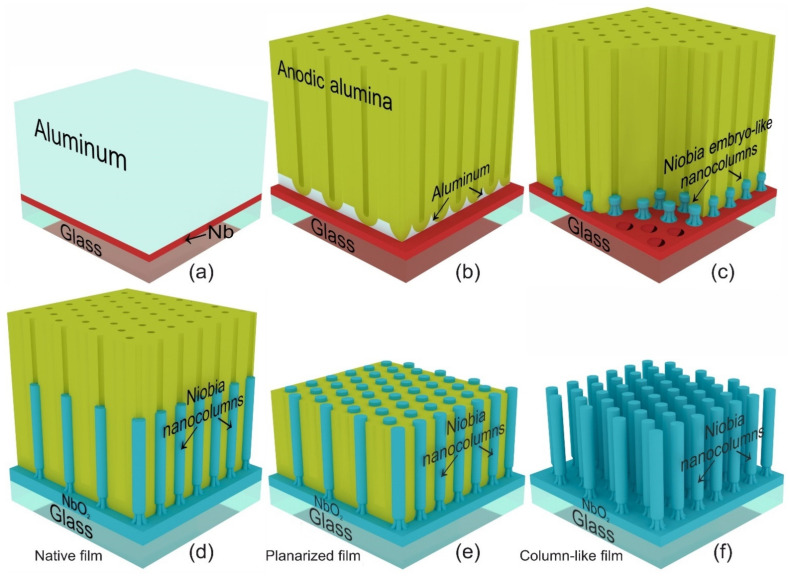
Three-dimensional schematic view showing the main steps of three type niobia nanostructured films production via porous alumina assisted anodizing of systems Al/Nb on glass substrate: (**a**) sputter deposition of Al/Nb bilayer; (**b**) anodizing the Al layer to form porous alumina film; (**c**) porous alumina assisted anodizing of the Nb layer; (**d**) porous alumina assisted high voltage reanodizing of the Nb layer on glass substrate to grow niobia (first type, native film); (**e**) planarization of niobia film by removal of the unfilled porous alumina portion (second type, planarized film); (**f**) completely removal of the porous alumina (third type, column-like film).

Three types of niobia nanostructured films were produced by PA-assisted anodizing of bilayer Al/Nb systems deposited on polished glass substrate with a thickness of 1070 µm and size of 6 × 9 cm^2^. The 3-D schematic view of nanostructured films production is shown in Figure 1.

**Figure 2:**
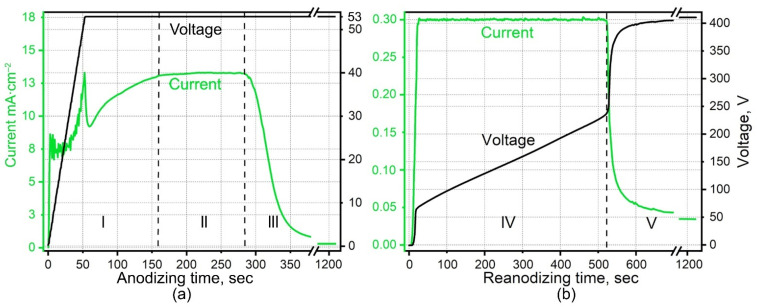
The voltage- and current-time responses during the porous alumina assisted anodizing (**a**) in 0.2 M oxalic electrolyte followed by reanodizing (**b**) in 0.5 M boric acid and 0.05 M sodium tetraborate electrolyte of an Al (1000 nm)/Nb (50 nm) bilayer system sputter-deposited on a polished glass substrate. The marked stages are: I—nucleation and beginning of a steady-state growth of pores in the alumina layer, II—steady-state pore growth (at 53 V) during the anodizing (Figure 1b), III—aluminum complete oxidation, nucleation, and development of niobia embryos (Figure 1c), IV—galvanostatic reanodizing the Nb underlayer to 230 V (Figure 1d), V—galvanodynamic-potentiostatic polarization at 410 V (current decay). The profiles have been presented in two separate panels, for the sake of clarity.

A layer of niobium, 50 nm thick, followed by a layer of aluminum, 1000 nm thick, were successively deposited on the substrates by the magnetron sputtering of Nb (99.95%) and Al (99.999%) targets as shown in Figure 1a. The glass substrates with Al/Nb were cut into samples with 2.8 cm^2^ area and anodized by a two-electrode scheme in a specialized cylindrical electrochemical cell with a horizontal anode, made of polytetrafluoroethylene [63]. The cell design allows one to get rid of meniscus effects during the anodizing process. The sample preparation process did not include any additional measures to increase the regularity of the films structure. The Al/Nb system was first anodized in a 0.2 M aqueous solution of oxalic acid in a potentiostatic mode at a 53 V (Figure 1b,c). Then, it was reanodized in an electrolyte containing 0.5 M boric acid and 0.05 M sodium tetraborate in a potentiodynamic mode by raising the voltage to 230 V. The first type of film—native film (NF)—is shown in Figure 1c. Figure 2 displays the voltage- and current-time responses of anodizing and reanodizing. To obtain the second type—planarized film (PF)—the PA part was smartly dissolved in the hot mixture of phosphoric and chromic acids (hereafter, the selective etchant [112]) for 476 s while maintaining a temperature of 50 °C as shown in Figure 1e. The third type—column-like film (CF) (Figure 1f)—was obtained by a complete removal of PA.

The temperature was maintained with the highest possible accuracy at 293 K and not exceeding ±1 K. Acids and sodium tetraborate were supplied by the Belaquilion additional-liability company and manufactured by Sigma-Aldrich, Inc (St. Louis, MO, USA). A programmable power supply 5751 A (Keysight Technologies Inc., Santa Rosa, CA, USA) was used as the anodizing unit, controlled by a personal computer with homemade software written in LabVIEW 2018 (National Instruments Corp., Austin, TX, USA). Programmable digital multimeters 34,470 A (Keysight Technologies Inc., Santa Rosa, CA, USA) were used to record the voltage–time responses, controlled by a personal computer with homemade software written in LabVIEW 2018.

### 2.2. Film Characterization

The NFs were observed in a SEM S-4800 (Hitachi High-Technologies Corp., Tokyo, Japan) operated at 10–15 kV, after coating the specimens with a thermally evaporated 3 nm thick gold layer to reduce the charging effects.

### 2.3. Optical Measurements

UV-VIS–NIR optical characteristics were measured on a Spectrophotometer MC–121 (SOL Instruments Ltd., Minsk, Belarus) with single-beam optical design and dual monochromator. The spectral slit width of the monochromator was fixed and amounted to 2 mm. The spectral scanning step was 2 nm in the range from 190 to 1100 nm at an incident angle of 10°. The optical measurements were carried out in the following sequence. Firstly, reflectance was measured. Then, transmittance measurements were performed. All light that did not hit the receiver was added to absorbance, including reflectance. Therefore, to obtain absorbance from the measured values, the previously obtained reflectance should be subtracted. In this case, according to the conservation law of energy, the sum of reflectance, transmittance, and absorbance is 100% and absorbance included light scattering. Based on the experimentally determined spectral dependences of transmittance and reflectance, the absorbance was calculated as:

(1) A=1−(T+R)

where *A*, *T*, and *R* are absorbance, transmittance, and reflectance, relative units.

The effective refractive index based on the optical spectra presented below and taking into account the first type of *NF* thickness (Figure 1d) was calculated using the following relation [113]:

(2) $${2h}_{NF}\cdot n\cdot \cos\theta =m\cdot \lambda$$

(3) $$h_{NF}\cdot n\cdot \cos\theta =\frac{2m+1}{4}\lambda$$

where *h_NF_* is the *NF* thickness, nm; *n* is effective refractive index; *θ* is incidence angle of the light beam, degrees; *λ* is wavelength, nm; *m* is order of interference.

### 2.4. Data Operation and FDTD Simulations

Graphical dependencies and curve-fittings were developed using OriginPro 2018 (OriginLab Corp., Northampton, MA, USA), and calculations are performed using mathematical tables Excel 2016 (Microsoft Corp., Mountain View, CA, USA).

The finite-difference time-domain (FDTD) simulations were performed with available software FDTD Solutions (Lumerical Inc., Vancouver, BC, Canada) on a laptop Inspiron 15 3576–8226 (Dell Tech., Round Rock, TX, USA). The area of simulations was set to 1 periods in the X-direction, 0.7 periods in the Y-direction, and 0.5 periods in the Z-direction for mode source. The dielectric material properties, permittivity:

(4) $$\sqrt{\varepsilon }=n+i\cdot k$$

where n+i·k is the complex refractive index, were taken from measurements carried out in ref. [114] for the 0.25–2.50 µm wavelength region as the default setting. A total field scattered source (TF-SF) was used for the simulation.

## 3. Results and Discussion

### 3.1. Anodizing Behavior

It should first be noted that the process of reanodizing the niobium sublayer on a glass substrate is carried out for the first time. In the work [115], some results are presented, but there are no voltage- and current-time responses and detailed results interpretation. Every result presented earlier [63,116,117,118] describes processes on silicon wafers with either additional conducting layers, or with a sufficiently thick niobium sublayer that does not completely reanodized and plays the role of a conducting layer itself. In the presented case, the thickness of the niobium sublayer is chosen so that it is completely transformed into oxide during reanodizing. This was necessary, firstly, to assess the possibility of such an approach, and, secondly, to improve the optical characteristics of the films, in particular, the optical transmittance in regions where there is no photonic band gap.

The choice of the anodizing mode was made from the following considerations. Oxalic electrolyte [42,119,120,121] is one of the best studied organic, economic, and environmentally friendly solutions for the formation of PA. The formation voltage of 53 V is a compromise, since it makes it possible to obtain nanocolumns with a sufficient thickness and high degree of ordering, verticality, and reproducibility. In addition, the formation mode of columns at 50–53 V in the oxalic electrolyte is well studied, since it was repeatedly used to obtain nanocolumns of tantalum [112,122,123,124], niobium [117,125], hafnium [126,127], zirconium [128].

**Figure 3:**
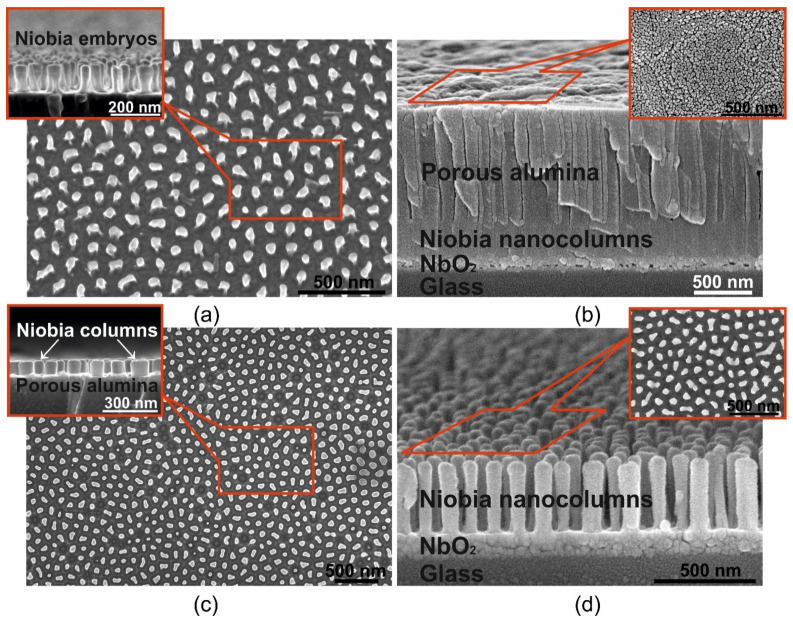
(**a**) SEM images of niobia nanostructured films formed after porous alumina assisted anodizing of Al/Nb bilayer system on glass at 53 V in 0.2 M oxalic acid and a complete removal of the porous alumina; (**b**) porous alumina assisted high voltage reanodizing in the mixed solution of 0.5 M boric acid and 0.05 M sodium tetraborate at 230 V (native film); (**c**) planarization of niobia nanostructured film by removal of the unfilled porous alumina portion (planarized film); (**d**) complete removal of the porous alumina (column-like film).

Figure 2 shows the electrochemical responses during the constant-voltage anodization of the Al/Nb/Glass sample in 0.2 M oxalic electrolyte. At the commencement of the formation, voltage rises at a rate of about 1 V·s^−1^ before reaching 53 V constant-voltage polarization, then the current reaches a value of 13.3 mA cm^−2^ and enters the stationary section after about 150 s (the end of stage I, Figure 2a). During the next stage II, a PA layer grows until its barrier layer reaches the underlying Nb metal in about 280 s at the given voltage and film thickness; then in the III stage, the current density begins to reduce, at an extremely fast rate of ~250 μA·s^−1^. Over time, the rate of fall decreases significantly. The aluminum grid (Figure 1b) is completely oxidized due to the current supply through the niobium sublayer. The nucleation and growth of niobia nanocolumns occurs (Figure 1c). Anodizing stops when the current reaches ~54 μA·cm^−2^. A drop in current to this value ensures aluminum grid complete oxidation and the formation of embryos niobia nanocolumns with morphological and electrophysical parameters necessary for further reanodizing. Figure 3a shows SEM images of niobia embryo-like nanocolumns after PA removal.

The voltage–time and current–time responses for reanodizing to 230 V of the Al/Nb/Glass samples are shown in Figure 2b. An initial potential surge of about 61 V is evident due to the presence of anodic niobia embryo-like nanocolumns under the pores. Keysight Power Supplies was able to support the power quite evenly without manual adjustment for a uniform raise of the reanodizing voltage (stage IV). Thus, the rise in the formation potential during the constant-current reanodizing confirms the field-assisted growth of anodic niobia while the final current decay, without overshoots and substantial fluctuations, is a good sign of completing the ionic transport through the anodic film at the established field strength. The voltage increase at constant-current would continue indefinitely in the case of an unlimited niobium sublayer thickness. In the case of a 50 nm thickness, the voltage sweep ends after 525 s at the 230 V mark (stage V). A very rapid rise in voltage begins, indicating that Keysight Power Supplies seems to feel that the voltage of 230 V is not enough to maintain the set current. The point is that the entire niobium sublayer is oxidized and the ionic resistance increases significantly. Forced voltage limiting at 410 V allows to curb explosive growth and protection the film from destruction. At this time, the value of the current continues to fall, the rate of falling slows down significantly over time. It is important to note that, despite the oxide nature, niobium dioxide NbO2 is a good conductor [63], which, after complete acidification of the layered metal film, takes on the role of a conductor to unoxidized regions of niobium, which can form due to imperfect uniformity and planarity of magnetron sputtering.

### 3.2. Films Morphologies

Figure 3 shows SEM images of nanostructured films at different stages of production. Figure 3a shows SEM images of niobia nanostructured films formed of the PA assisted anodizing bilayer system Al/Nb on glass at 53 V in 0.2 M oxalic acid and a complete removal of the PA (a schematic 3-D view of this structure, but with PA is displayed in Figure 1c). Figure 3a shows quasi-regularly spaced embryo-like nanocolumns of anodic niobia, oriented perpendicular to the sample surface. Each embryo begins to form when the bottom of the alumina pore reaches the surface of the niobium sublayer. Attention should be paid to the uniformity of the embryos. All embryos have almost the same height and diameter. This indicates that the anodizing front and various oxide cells of the PA reach the niobium sublayer simultaneously. This provides a high degree of uniformity of the niobia nanocolumns formed later during reanodizing from these embryos and the regularity of the planarized structure or a structure consisting of free-standing nanocolumns of anodic niobia.

Figure 3b demonstrates the film after PA assisted high voltage reanodizing in the mixed solution of 0.5 M boric acid and 0.05 M sodium tetraborate at 230 V (native film, on 3-D model corresponds to Figure 1d). According to the SEM image, it was determined that the total thickness of the NF is 1408 nm, while the thickness of the alumina pores not filled with the niobia nanocolumns is 962 nm, therefore, the height of the alumina pores filled with the niobia is 325 nm and the sublayer of the continuous niobia NbO_2_ has a thickness of 115 nm. Thus, the NF is a three-layer structure: PA/PA filled with niobia/a continuous niobia layer of composition NbO_2_ [117], which is located on the surface of the glass substrate.

Figure 3c shows the surface and cross section SEM images of PF (a schematic 3-D view of PF is displayed in Figure 1e). The top view shows the almost complete absence of defects in the formed structure. This means that absolutely all formed embryos stretched out into the alumina pores during reanodizing and turned into niobia nanocolumns. Figure 3c shows that the column height is extremely uniform—all the tops of the anodic niobia nanocolumn uniformly protrude above the PA surface, and the PA surface is characterized by a high degree of planarity. PA residues are not observed, which may indicate correctly selected planarizing etching regimes. As can be seen from Figure 1e and Figure 3c, the PF is a three-layer structure. The first layer is the protruding vertices of niobia nanocolumns with a height of 90 nm. The second layer with thickness of 235 nm is a PA with niobia nanocolumns located in the pores of 65 nm in diameter at a distance of 125 nm. The third layer is a niobia continuous layer NbO_2_.

Finally, the third type—CF of the studied samples, which is a column-like structure consisting of free-standing niobia nanocolumns with completely removed PA—is shown in Figure 3d (a schematic 3-D view of CF displayed in Figure 1f). The cross-section SEM-images of CF shows that the PA template, which sets the morphological parameters of the column-like structure during its formation, is really removed without a trace; at the same time, free-standing columns retain their orientation along the normal to the sample surface. In the inset, which is a top view of such a structure, it is clearly seen that all the nanocolumns are level, there are no “fallen” or tilted nanocolumns, and the structure is characterized by the same degree of regularity and defect-freeness as for the embryos shown in Figure 3a and PF shown in Figure 3c. The figure shows that the thickness of the NbO_2_ layer is 115 nm. According to the data of electron microscopic studies, the diameter and height of the columns are equal to 65 and 325 nm, respectively.

### 3.3. Film Optical Characterictics

**Figure 4:**
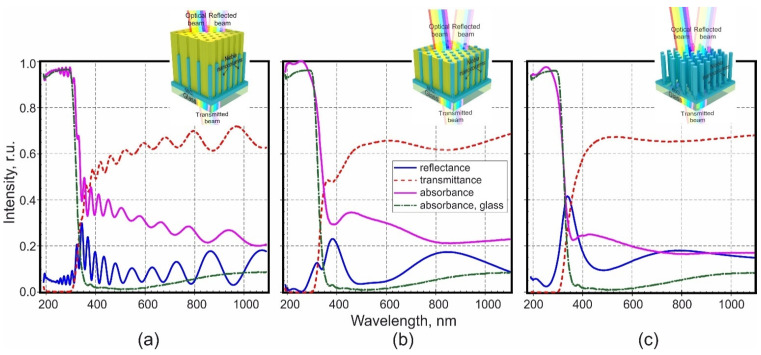
Optical characteristics of three types of niobia nanostructured films: (**a**) native film, (**b**) planarized film, (**c**) column-like film and glass substrate.

In the work, the reflectance and transmittance spectra of the three type’s films were investigated. Based on the experimentally determined spectral dependences of transmittance and reflectance, the absorbance including light scattering was calculated (Equation (1)). The spectral characteristics of the three types of films are shown in Figure 4, 3-D schematic views of the studied films and the experiment diagram are presented in the inserts. For comparison, the spectra of all films show the spectral dependence of the absorbance of the glass substrate (green dash-dotted line).

#### 3.3.1. Native Films

In Figure 4a the spectral characteristics of the NF are shown, which is a three-layer structure located on a glass substrate. The first two-layers (PA and PA filled with niobia) have a nanoscale structure with a periodically changing refractive index themselves. The third layer is a thin continuous niobia IV. If abstract from the intrinsic spectral-optical characteristics of the glass substrate, then several factors will affect the propagation of light in such an environment:

1. The presence of a nanoscale periodic structure in the first two-layers;
2. Intrinsic absorbance of light by at least three different materials—alumina, niobia V and IV, and, most likely, non-stoichiometric niobia in the transition regions;
3. Interference of light reflected from the interfaces of different layers.

The abovementioned complicates the consideration and interpretation of the spectra.

The dynamics of transmittance in the ultraviolet range shows that at wavelength of 190 to about 300 nm, almost 97% absorbance occurs, and transmittance and reflectance are negligible. A further increase in wavelength leads to decrease in absorbance and increase in reflectance and transmittance. Moreover, all the curves have pronounced oscillations absorbance and transmittance beginning with ~360 nm, reflectance ~250 nm, which indicates the presence of boundaries between the environments and Fabry–Perot interference. The following boundaries can be distinguished: air/PA, PA/PA filled niobia, PA filled niobia/continuous layer NbO_2_, continuous layer NbO_2_/Glass, Glass/Air. Two peaks can be distinguished on the reflectance curve, masked by the interference pattern: a peak in the short-wavelength region with maxima at 355 nm and a peak in the long-wavelength region, presumably at about 950 nm. The authors believe that the presented interference pattern is due to the reflectance of light at the air/PA and continuous layer NbO_2_/Glass interfaces. The interference pattern served as the basis for calculating the effective refractive index (see Section 3.4 and Section 3.7).

#### 3.3.2. Planarized Films

The PF is built of two layers of different thickness with a periodically varying refractive index as shown earlier in Figure 1e (3-D view) and Figure 3c (SEM image), and the third layer, as in the previous case, which is a thin continuous niobia IV. The spectral characteristics of PF are shown in Figure 4b. As can be seen from Figure 4b, the presence of such a two-layer structure led to the existence of several distinct maxima in the reflectance curve at about 320 and 380 in the short-wave region and one band at around 850 nm in the long-wave region. Interpretation and modeling of the optical properties of such a structure is a rather difficult task, but the authors believe that the indicated maxima on the reflectance curve are the result of the interaction of light with the indicated two PF layers with periodically changing refractive index.

#### 3.3.3. Column-Like Films

Column-like structure (Figure 4c) is a single 325 nm thick layer with a periodically changing refractive index, also resting on a continuous thin layer of NbO_2_, and in this case, the difference in refractive indices (air and niobia) is very significant. The reflectance spectrum of such a structure clearly shows a pronounced peak in the short-wavelength region of the spectrum with an intensity of more than 40% with a maximum at about 340 nm and an extended peak of low intensity in the long-wavelength region with a maximum at 790 nm.

The authors consider that a periodic change in the refractive index in the structures under the study is responsible for the appearance of photonic band gaps. The proximity of the location of the maxima in the reflectance spectra of three different films is due to the almost complete identity of the main morphological parameters of the nanostructured films, and the reason for some differences in the spectra is the difference in refractive indices for different films and the features of the structure and composition of each of the studied films. Improving the regularity of the nanocolumn position and some other measures will significantly increase the reflectance efficiency. One of the proposed measures could be such a modification of the morphology of the described films, which would make it possible to shift the maximum located in the short-wave region towards longer waves, where the absorbance of light by the substances that make up the films would be less. This, in turn, would increase the structure efficiency as PC.

To test this assumption, in Section 3.6, a FDTD simulated PC using the example of the simplest structure—CF—is presented.

### 3.4. Calculation and Discussion of Spectral Dependence of Native Film Refractive Index

Unfortunately, optical transmittance, reflectance, and absorbance spectra (shown in Figure 4a) for NF show strong Fabry–Perot interference masking the intrinsic optical properties of the structure materials—absorbance bands, for example. At the same time, due to the presence of a clearly defined interference pattern, it becomes possible to calculate the effective refractive index of the film as a whole. If performing calculations based on the reflectance spectrum, then the influence of the glass substrate is completely excluded, since, due to its significant thickness, it does not contribute to the interference pattern in the investigated frequency range, and thus, the interference pattern is formed due to light reflected from the interface Air/PA and niobia IV surface/Glass. Due to the closeness of the NF thickness and the light wavelength in the optical range, the spectral characteristics of such films are characterized by the Fabry–Perot interference. Using the interference pattern, the effective refractive index of the NF was calculated, which in the investigated wavelength range 190–1100 nm was 1.54–1.75. Further calculations of the refractive indices of the components that make up the NF, which are discussed below, have shown that the results are in satisfactory agreement with the literature data (see Section 3.5).

**Figure 5:**
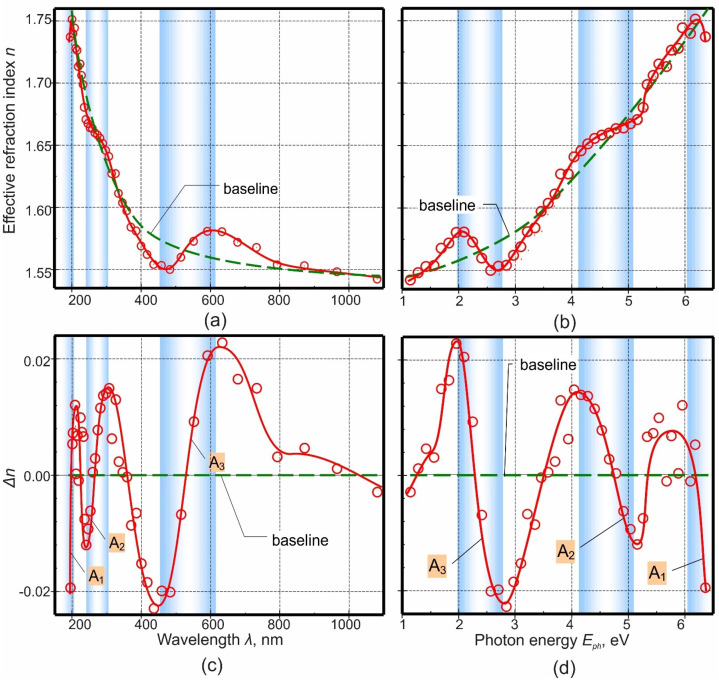
Dependence of (**a**,**b**) effective refraction index *n* and (**c**,**d**) dispersion Δn on wavelength and photon energy for native films. A_1_, A_2_, A_3_—areas of anomalous dispersion with assumed absorption bands are in the energy ranges of 6.0, 4.2–5.1, 1.9–2.8 eV, respectively.

Figure 5a shows the dependence of the effective refractive index *n* on the wavelength, calculated from conditions (2) and (3) and based on the reflectance spectrum of the NF shown in Figure 4a. The NF thickness is such that it causes the appearance of an interference pattern in the entire investigated spectral range, with a large number of extrema. This means that the dispersion dependence n=f(λ) shown in Figure 5a was constructed using a large number of experimental points and its shape is reliable.

When considering the dispersion curve (Figure 5a, red solid line), first of all, the presence of a wide maximum in the wavelength range of 440–850 nm attracts attention. In addition, upon closer examination of the short-wavelength part, regions with a periodically varying rate of n=f(λ) and a small portion of the refractive index growth with increasing wavelength in the region close to 200 nm are visible. This character of the dispersion curve can be explained by a combination of several reasons. Firstly, the system under study is multicomponent and, at least, contains three different chemical compounds: PA, which is amorphous [43,129,130], niobia V (nanocolumns embedded in the PA), and niobia IV (sublayer located on the glass surface and on which nanocolumns stand), which are also amorphous [117,131]. The state of niobia is not so unambiguous, since, for example, field crystallization of niobia V is possible, stimulated by the presence of mechanical stresses (the compressive stresses in the amorphous anodic niobia that can facilitate crystal growth), which are guaranteed to be present during the growth of niobia nanocolumns into the alumina pores [132]. It is also clear that there cannot be a sharp interface between niobia IV and V, and there certainly is a non-stoichiometric transition region that is a mixture of oxides and has a sufficiently large thickness. Least, nanocolumns of higher niobia and PA are doped with impurities embedded from the anodizing electrolyte [117,131,133,134,135]. As a result, the total value of the effective refractive index is determined by the contribution of each of the listed components, and none of them can be neglected due to their comparable relative amount and significant difference in refractive indices.

Further, when considering the dispersion curve, the authors were guided by the well-known fact that the normal dispersion at the wavelengths corresponding to the absorbance bands is replaced by the so-called anomalous dispersion. An increase in the wavelength (a decrease in the frequency of optical radiation) leads not to a decrease, but to an increase in the refractive index. This phenomenon [136] is not something new, it was discovered for the first time in the second half of the 19th century and is considered in textbooks on optics, for example, in ref. [137]. An explanation for it was given later, and at present, this phenomenon serves both as a means of studying the properties of materials and is used in photonics [138,139].

Thus, the selection of areas with anomalous dispersion on the n=f(λ) curve makes it possible to estimate more or less accurately the position of the absorbance bands, which, are masked by the interference pattern in the corresponding spectra (Figure 4a). Areas of anomalous dispersion were identified using the OriginPro program. A detailed description of the determination method is presented in the Section S1 of the Supplementary Materials. There are three areas of anomalous dispersion: they are marked, designated, and numbered uniformly (A_1_, A_2_, and A_3_) in both groups of Figure 5a–d. These areas, as already mentioned above, correspond to light absorption bands, which are also marked in both groups of figures.

The assumed absorption bands are in the energy ranges: 1.9–2.8 (anomalous dispersion region A_3_), 4.2–5.1 eV (anomalous dispersion region A_2_), the assumed beginning of the next absorbance band is 6.0 eV (anomalous dispersion region A_1_), then the band extends into the region of even shorter wavelengths (high photon energies). The position of the edge of the last high-energy optical absorption band is not entirely reliable, since it is located at the very edge of the investigated spectrum, and its beginning is evidenced by a small number of experimental points. In addition, the authors also do not yet know how to interpret the noticeable scatter of points in the 5.6–5.8 eV region (no special marking). This can be caused both by random errors in determining the positions of the extrema on the interference reflectance curve and, accordingly, in calculating the refractive index, or by some inaccuracy in determining the position of the baseline, and by the presence of a weak absorbance band, caused, for example, by the complex shape of the allowed bands and the presence of possible transitions between bands.

### 3.5. Assignment of Oxide Absorbance Bands

An analysis presented in Section S2 of the Supplementary Materials makes it possible to classify the absorbance bands as follows:

1. The authors believe that the absorbance band starting at about 5.7 eV (wavelengths less than 200 nm, anomalous dispersion region A_1_ on Figure 5) and extending further into the region of even shorter wavelengths (high photon energies) is due to the alumina cellular-porous structure, its purest part.
2. The scatter of points in the range of 5.6–5.8 eV (no special marking) can be caused by both random reasons and errors in data processing, and insignificant optical absorbance with the participation of electronic states in the band gap of aluminum oxide, caused by disordering of the structure, impurities introduced from the electrolyte, or possibly by the presence of niobium ions dissolved in alumina.
3. Based on the analysis of a large amount of literature data, the authors believe that the absorbance band found in the range 4.2–5.1 eV (anomalous dispersion region A_2_ on Figure 5) may be due to the presence of niobia V, stoichiometric and pure, or with an impurity of Al_2_O_3_, or/and the most contaminated part of impurities anodic alumina.
4. The authors suggest that the absorbance band, which is in the range 1.9–2.8 eV (anomalous dispersion region A_3_ on Figure 5), may be due to the presence of mixed niobia of an indefinite composition that varies with respect to the height and diameter of the nanocolumns and the film thickness.

This kind of assignment is just a hypothesis and one of the possible interpretations of the results. Further studies will clarify the remaining questions regarding the optical properties of such structures. A more accurate and detailed study of CF requires the use of equipment capable of performing a step-by-step study of the composition and oxide phase of one nanocolumns with a step of several nanometers. In addition, special preparation of films is required so that nanostructuring does not affect the study process, and at the same time, the interference and intrinsic optical properties are not masked by size effects or an interference pattern. A clear understanding of the spectral absorbance regions and their origin, the presence and nature of allowed states caused by structural defects and impurities, would make it possible to create, for example, luminescent structures that additionally have their own photonic band gap.

### 3.6. 2-D Photonic Crystals

To test the applicability of the formed nanostructures as 2-D PCs and to evaluate their efficiency, their optical properties were simulated. For simulation, firstly, the simplest structure was chosen, which is a column-like array of nanocolumns of niobia V, free standing on a sublayer of niobia IV. Secondly, the simulation was divided into two parts, during which different tasks were solved. The first task was to reproduce the real reflectance spectrum of the CF as closely as possible by the model. The successful solution of the first problem allows choosing for the model the physical characteristics of materials that correspond to reality as much as possible, which would be confirmed in the maximum coincidence of the real and model spectra. The second task was to select such morphological parameters that would make it possible to increase the efficiency of CF as a PC. The solution of the second task gives the optimal morphological parameters for the development of PC with maximum efficiency. The conditions for simulating concerned both the morphological parameters of the structure (the height of the columns, diameter, the distance between the columns, the nature of the packing of the structure) and the physical characteristics of the materials: the values of the refractive indices and extinction coefficients; moreover, when setting these parameters, it was important to take into account their dependence on the wavelengths. The initial morphological parameters for modeling the real reflectance spectrum were determined as a result of electron microscopic studies of the real CF and are shown in Table 1.

**Figure 6:**
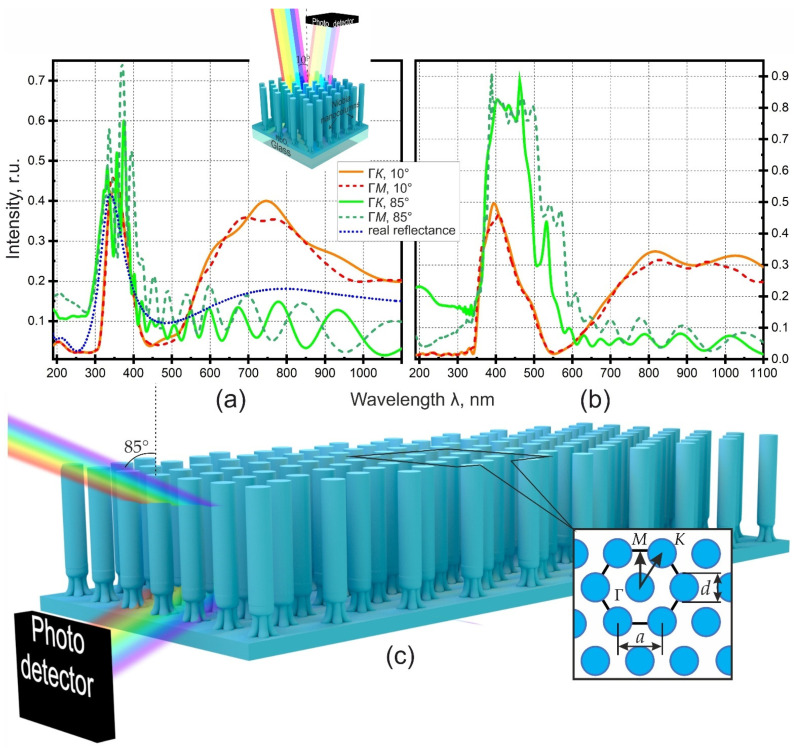
FDTD Simulation of optical reflectance of a (**a**) column-like film and (**b**) 2-D photonic crystal at different angles of incidence (10° and 85°) and directions of propagation (Γ*M* and Γ*K*) of an optical beam. (**c**) Three-dimensional schematic view of 2-D photonic crystal created in Lumerical FDTD Solutions.

Based on the generally accepted models of the structure of PA [53,55,68,74,120,140,141,142,143,144,145,146,147], it was assumed that the columns of anodic niobia formed inside the pores are hexagonally arranged on the film surface. The spectra calculated for the model structures are shown in Figure 6.

Any ordered structure, including hexagonal packing, implies anisotropy, which is always taken into account when considering and modeling the optical properties of such structures [55,69,89,98,100,108]; therefore, spectra were obtained for two nonequivalent directions of the incident ray Γ*K* and Γ*M* (explanation on the callout of Figure 6c) relative to the unit cell of an idealized two-dimensional structure. At the same time, it should be taken into account that a real structure is actually packed “amorphously” and is isotropic in the film plane due to the presence of a domain structure or short-range order of the oxide cells [44,68,144,148,149,150]. Therefore, the model spectra constructed on the basis of the concept of ideal hexagonal packing, in any case, cannot ideally coincide with the experimental results. The selection of the physical characteristics of the materials that make up the real CF presented a particular difficulty in solving the first problem. This is due to both the scarcity of information and its weak applicability to anodic oxides, which was discussed above. Selecting refractive index of FDTD simulation are presented in the Section S3 of the Supplementary Materials.

As a result, by trial and error, taking into account the literature data, the parameters of the materials were selected, which made it possible to achieve an acceptable similarity between the model reflectance spectra (Figure 6a) and the real spectrum obtained for the CF sample (blue dotted line). Two groups of model spectra were constructed, differing in the angle of incidence of the light beam. At an angle of incidence of 10°, the spectral lines corresponding to the directions Γ*K* and Γ*M* are very close and in the short-wavelength region differ little from the experimental dependence. A difference is observed between the experimental and calculated dependences in the long-wavelength region. The impossibility of taking into account the increase in the absorbance of light by the sublayer of niobium dioxide in the region of long wavelengths did not make it possible to achieve a decrease in the intensity of the broad peak in the region of 550–950 nm. 

The second group of model spectra was also calculated for both directions of the beam relative to the unit cell of the idealized structure, but the angle of incidence was increased to 85°, and the photodetector was placed at the end of the film as shown in Figure 6c. In this case, the differences between the spectral dependences calculated for different directions Γ*K* and Γ*M* are more significant, but the calculated reflectance efficiency in no case exceeds that actually achieved in the experiment.

After optimizing the optical characteristics of the materials, the second task was completed: with the fixed values of the refractive indices and extinction coefficients, the morphological parameters of the CF were manually selected according to the criterion of the maximum reflectance of the incident light. The selection results are also presented in Table 1, and two groups of calculated spectral dependences are shown in Figure 6b. It is noted first that the reflectance efficiency is higher for 85° beam, reaching about 92% for the Γ*K* direction at a wavelength of 462 nm. A sharp increase in the reflectance efficiency became possible due to the shift of the reflectance maximum to a longer wavelength region, in which there is no absorbance of light by materials of CF, as a result of optimization of the morphological parameters (geometry) of the FDTD simulated nanocomposite material. The anisotropy of the model structure does not significantly influence the optical spectra—at any angle of incidence, the shape of the reflectance curves of light directed along both directions (Γ*K* and Γ*M*) are similar in shape. At the same time, the reflectance efficiency depends very much on the angle of incidence. If at an angle of incidence of 10°, the reflectance only approaches 50%, then an increase in the angle of incidence to 85° leads to an increase in reflectance efficiency to 92%.

Thus, as a result of simulating the 2-D PC, the optimal parameters of its morphology were determined. A further direction of research is the selection of methods for fabricating structures similar to the calculated one and fitting the optimal parameters based on experimental data.

It should be noted that the maximum reflectance efficiency achieved in FDTD simulation can probably only occur in anisotropic, highly ordered structures. At the same time, the spectral characteristic of the reflectance has some, albeit insignificant, incident beam dependence on the direction. In the case of random orientation or in the case of an isotropic structure in the sample plane (in the arrangement of the columns there is only a short-range order), the reflectance will have an intermediate spectral characteristic. Therefore, in addition to achieving optimal morphological parameters, measures should be taken to maximize structure ordering, maximize anisotropy, and to achieve the desired orientation of the structured sample relative to the incident beam in order to achieve the necessary spectral characteristics of reflectance.

### 3.7. Native Film Refractive Index

In this work, the effective refractive index of an NF sample, consisting of three layers of oxide materials, was calculated. The calculation result for anodic alumina with unfilled pores was 1.56, and for a layer with pores filled with Nb_2_O_5_ was 1.72.

To calculate the effective refractive index of laminar structures, the work provides two formulas for the cases if the electric field is orthogonal to the direction of the structure stratification and electric field is along the direction of the structure stratification. For the first case, the calculated refractive index was 1.68, and for the second, 1.63. A detailed description of the calculations is presented in Section S4 of the Supplementary Materials.

## 4. Conclusions

Three types of niobia nanostructured films produced on glass substrate with a thickness of 1070 µm and size of 6 × 9 cm^2^ were formed by porous alumina assisted anodizing, first in a 0.2 M aqueous solution of oxalic acid in a potentiostatic mode at a 53 V, then by reanodizing the material in an electrolyte containing 0.5 M boric acid and 0.05 M sodium tetraborate in a potentiodynamic mode by raising the voltage to 230 V. Anodic behaviors, morphology, and optical properties of the films have been investigated. FDTD simulation was carried out and the morphology of a potential 2-D photonic crystal, which was designed based on the third type of films, was proposed. As a result of the work, the following points have emerged:

1. Smart anodic processing of a two-layer Al/Nb system with a thickness ratio of 1000/50 nm makes it possible to completely acidify metal layers and form nanostructured films with a high degree of transparency of about 70% for all types of films obtained.
2. Three types of films located on a glass substrate were obtained: native, planarized, and column-like.
  2.1. Native film is a three-layer structure with the total thickness of 1408 nm, while the thickness of the not filled porous alumina is 962 nm, the height of the alumina pores filled with the niobia is 325 nm, and the sublayer of the continuous niobia NbO_2_ has a thickness of 115 nm.
  2.2. The planarized film is also built of three layers. The first layer is the protruding vertices of niobia nanocolumns with a height of 90 nm. The second layer with 235 nm thickness is a porous alumina with niobia nanocolumns located in the pores of 65 nm in diameter at a 125 nm distance. The third layer is a niobia continuous layer NbO_2_.
  2.3. A column-like film consisting of full free-standing niobia nanocolumns with a height of 325 nm, diameter of 65 nm and a distance of 125 nm also resting on a continuous thin layer of NbO_2_.
3. The spectral characteristics of the film three types in near and middle UV, visible and near IR ranges have been investigated. The native film spectra are characterized by the Fabry–Perot interference, which is determined by film thickness. The reflectance spectra of all films show peaks in short- and long-wave regions. The presence of the peaks can be explained by a multi-layer composition of the films with a periodically changing refractive index in two dimensions.
4. The interference pattern served as the basis for calculating the effective refractive index of native film, which varies within 1.75–1.54 in the wavelength range 190–1100 nm. The spectral characteristics of the refractive index show areas of normal and anomalous dispersion. The latter made it possible to distinguish a number of absorption bands of the native sample.
5. Based on the analysis of a large amount of literature data, the identified oxide absorbance bands were assigned.
  5.1. The optical absorbance band of native film starting at about 5.7 eV (wavelengths less than 200 nm) and extending further into the region of even shorter wavelengths (high photon energies) is due to the alumina cellular-porous structure, its purest part.
  5.2. The scatter of points in the range of 5.6–5.8 eV can be caused by insignificant optical absorbance with the participation of electronic states in the band gap of alumina, caused by disordering of the structure, impurities introduced from the electrolyte, or possibly by the presence of niobium ions dissolved in alumina.
  5.3. The absorbance band of native film found in the range 4.2–5.1 eV may be due to the presence of niobia V, stoichiometric and pure, or with an impurity of Al_2_O_3_, or/and the most contaminated part of impurities anodic alumina.
  5.4. The absorbance band of native film, which is in the range 1.9–2.8 eV, may be due to the presence of mixed niobia of an indefinite composition that varies with respect to the height and diameter of the nanocolumns and the film thickness.
6. The value of the effective refractive index of the native film according to the existing effective-medium models (Maxwell–Garnett model and model of the laminar structure) was calculated to be in the range of 1.63–1.68.
7. The optical characteristic of the third type of the studied film with column-like structure was used in FDTD simulation to obtain the morphology of a potential 2-D photonic crystal with 92% (wavelength 462 nm) reflectance: 80 nm diameter, 700 nm height, 200 nm inter-column distance, 150 nm continuous NbO_2_ thickness.

Niobia column-like 2-D photonic crystals will find wide application in nano-optics and nano-photonics. It can be built into an optical waveguide scheme similar to that considered in ref. [101], with the difference that the cylindrical elements pores will be not filled with air (a dielectric constant lower than the base material) but with anodic niobia.

## Figures and Tables

**Table 1:** Morphological parameters of real (experimental) column-like film and FDTD simulated 2-D photonic crystal.

Morphological Parameters	Column-Like Film (Real)	2-D Photonic Crystal (FDTD Simulated)
Column diameters, nm	65	80
Column heights, nm	325	700
Distance between column centers, nm	125	200
Continuous NbO_2_ thickness, nm	115	150
Reflectance, %	42	92
Wavelength, nm	340	462

## Data Availability

Not applicable.

## Supplementary Materials

The following are available online at https://www.mdpi.com/article/10.3390/mi12060589/s1: Table S1. Initial data and calculation results of the native film effective refractive index; Section S1. Determination of Anomalous Dispersion Areas; Section S2. Analysis for Oxide Absorbance Bands; Section S3. Selecting Refractive Index of FDTD Simulation; Section S4. Calculation of Native Film Refractive Index.
