# Supplementary material for: Optical Properties of Porous Alumina Assisted Niobia Nanostructured Films–Designing 2-D Photonic Crystals Based on Hexagonally Arranged Nanocolumns
Source: Micromachines (Basel). 2021 May 21;12(6):589. doi: 10.3390/mi12060589 (PMC8223973; doi:10.3390/mi12060589)
Supplement: Supplementary file 1 [file micromachines-12-00589-s001.zip › micromachines-1204991-supplementary.pdf]

micromachines

MDPI

Supplementary Materials

Optical Properties of Porous Alumina Assisted Niobia Nanostructured Films—Designing 2-D Photonic Crystals Based on Hexagonally Arranged Nanocolumns

S1. Determination of Anomalous Dispersion Areas

Firstly, the wavelengths λ according to the well-known equation from ref. [1]

$$E_{ph} = \frac{1.24}{\lambda} \quad \text{(S1)}$$

were recalculated in the photon energy E_{ph}, eV, and plotted the dependence $n = f(E_{ph})$, shown in Figure 5b. Using the OriginPro program for the dependence $n = f(\lambda)$, the baseline was first built automatically, then manually adjusted so that the resulting baseline was as "smooth" as possible. The number of points on the baseline was chosen to be 50 also in order to increase the number of baseline segments and reduce their length. Then the baseline was automatically built for the dependence $n = f(E_{ph})$ and the coordinates of the previously obtained baseline for the baseline dependence, which were appropriately recalculated along the abscissa axis, were added to the data table for the baseline in these coordinates. A manual adjustment (operation "modify") of the thus obtained $n = f(E_{ph})$ baseline was also carried out. Then, after recalculating the coordinates along the abscissa axis of the fitted data, they were again entered into the table for constructing the baseline for the dependence $n = f(\lambda)$ and re-fitted. Several iterations of the same kind were carried out so that the resulting curves in both coordinate systems $n = f(\lambda)$ and $n = f(E_{ph})$ looked perfectly smooth. After that, the "subtract" of the baseline was performed from the real graphs for both dependences $n = f(\lambda)$ and $n = f(E_{ph})$, and the results are shown in Figure 5c and 5d, respectively. As can be seen when considering Figures 5c and 5d, in both plots obtained after the "subtract" operation, areas of "normal" and "anomalous" dispersion coexist.

S2. Analysis for Oxide Absorbance Bands

When analyzing the literature data on the oxide electrical properties of interest, it should be borne in mind that the PA and niobia, which are parts of the formed nanostructured films, have a defective structure. In disordered materials, the boundaries of the allowed bands are blurred due to the appearance in the forbidden band of a large number of localized states, which leads to significant differences in the electronic structure of crystalline and disordered materials. A number of models exist and are being developed that describe the electronic structure of disordered materials [2–7]. This blurring of boundaries is often referred to as Urbach tails. Urbach tail is almost a universal property of disordered solids and Urbach tails in optical absorbance near band edges are observed and investigated in a wide variety of materials having disorder of several origins [8–11]. The assignment of absorbance bands is not an easy task due to the fact that comparison of data related to crystalline materials with structures obtained as a result of anodization is not entirely correct. The data on the optical characteristics and oxide band structure of interest are extremely inconsistent. This can be explained by the fact that, firstly, the position and width of the zones are determined by the crystalline modification of the oxide, and secondly, the degree of structural perfection, depends on

the amount of impurities and defects. All these features are determined by technologies and specific methods for the oxide formation, which are briefly discussed in the introduction. The variety of methods for the formation of oxide materials leads to a significant difference in properties that cannot be neglected. Quite a lot of works are known devoted to determining the band gap of amorphous metal oxides, including anodic ones (unfortunately, there are very few recent works [12,13]), which will be considered below. Let briefly consider the results known to the authors, grouped by the nature of the oxides.

S2.1. Alumina

It is known that Al_2O_3 can exist both in an amorphous state and in several crystalline modifications. All modifications differ in the ratio of tetrahedral and octahedral coordination of aluminum ions [14]. Regardless of the polymorphic modification, in ref. [15] for alumina, the range of values of the band gap of 7–9.5 eV is given. The most convenient for use in microelectronic technology are the amorphous phase (α-Al_2O_3) and the γ-phase (γ-Al_2O_3) [16]. Each modification is characterized by its own band gap: for the γ phase, the band gap lies in the range 7.1–8.7 eV [17], and for the amorphous phase, 5.1–7.1 eV [17]. It should be noted that the band gap depends on the synthesis method. Molecular layering (ML) allows one to obtain amorphous films with a band gap of 6.2 eV [18], while α-Al_2O_3 films grown by chemical vapor deposition (CVD) have a band gap of 5.6 eV [19]. In ref. [20], the value of band gap for amorphous alumina obtained by the ALD method is indicated, 7.0±0.1 eV, and in ref. [21] the results indicate that amorphous anodic Al_2O_3 formed in 20% sulphuric acid has a direct band gap of 7.3 eV, which is about 1.4 eV lower than its crystalline counterpart (single-crystal Al_2O_3). The optical band gap of sputter-deposited films was found to be in the range of 5.4–5.55 eV and that of evaporated film is 5.75 eV [22]. Anodic alumina investigated in [13] was obtained under the closest conditions in 0.3 M oxalic acid, but the band gap found for it is only 3.7 eV, which seems to be an extremely low value, even despite the obvious blurring of the boundaries of the allowed bands due to the emergence of Urbach tails. In the same work, for anodic alumina anodized in 0.3 M sulfuric acid, a band gap value of 4.3 eV is given. For PA elaborated on glass substrate there is no numerical data in ref. [23], only graphs. The potentiostatically anodizing of aluminum foil was carried out in a 10% sulfuric acid solution at 15 V at 288 K. Apparently, this is precisely the effective refractive index of the PA film, and it varies quite significantly for different films and wavelengths. The main thing is that anomalous dispersion is not observed on the graphs $n = f(\lambda)$ built for the interval 250–900 nm. Consequently, there are no absorption bands in this gap.

S2.2. Niobia V

The review [24] states that the band gap energy of niobia has been described to be within the ranges of 3.1 to 5.3 eV (semiconductor to insulator with conductivity). However, it is feasible to adjust the Nb_2O_5 band gap and variables like stoichiometry, heat treatment, crystallinity, and integrated external ions may be able to unusually impact the band gap energy, but source [15] gives a slightly narrower interval values 3.4–5.3 eV. In ref. [25] Nb_2O_5 thin films were deposited onto heated glass substrates by RF magnetron sputtering using a Nb_2O_5 target. The films were annealed in air at temperatures between 400 and 700 °C for 6 h. As the annealing temperature is increased from 400 to 700 °C the band gap of the film decreases to 3.60 from 3.74 eV. This trend contradicts the ref. [26]. For Nb_2O_5 films, the optical band gap is observed to increase from 4.35 eV when the films are in amorphous state to 4.87 eV on crystallization [26]. Authors ref. [27] as a result of optical studies of niobia films prepared by the reactive DC magnetron sputter process, the band gap changes from 3.58 eV for amorphous to 3.97 eV for crystalline Nb_2O_5 films at thicknesses of about 300 nm and the band gap changes from 3.51 to 3.86 eV for amorphous and crystalline films, respectively, for thicknesses of the order of 50

nm. In the literature review [28], Nb_2O_5 is indicated to be an *n*-type semiconductor with a band gap of about 3.4 eV, while, according to the results of the authors' own research for powders Nb_2O_5, were prepared by two different synthesis method, Sol-Gel and polymeric precursors (Pechini) ultraviolet-visible diffuse reflectance spectroscopy indicated a variation in the optical band gap values 3.32–3.40 eV in crystal growth process. It was measured in ref. [29] that the indirect optical band gap is ~ 3.65 eV for Nb_2O_5 films with a thickness of tens to several hundred nanometers, obtained by annealing deposited Nb films in an Ar flow and without oxygen plasma in a quartz case. According to ref. [30], the band gap values were between 3.15 and 3.25 eV. A band gap energy of 3.42 eV has been determined for polycrystalline Nb_2O_5 films, and 3.50 eV for amorphous Nb_2O_5 films [31]. This scatter of parameters is explained, in particular, by the fact that the optical properties of Nb_2O_5 are highly dependent on the sintering temperature [32]. In ref. [33], in detail the optical properties of Nb_2O_5 thin films by the sputtering method under different deposition parameters were investigated and the band gap takes values in the range 3.73–3.91 eV depending on the film deposition temperature and the oxygen content in the plasma, with the maximum value of band gap energy corresponded to the lowest (room) temperature and the maximum concentration (20%) of oxygen. Nb_2O_5, an important *n*-type oxide semiconductor, has a band gap in the range from 3.2 to 4 eV [34] depending on oxygen stoichiometry. In ref. [12], the spectral characteristics of the photocurrent for amorphous anodic Nb_2O_5 were estimated: the long-wavelength absorbance edge equal to 4.1 eV and the absorbance edge for direct dipole transitions with a value of 3.45 eV. The absorbance edge shift is related to the Urbach absorbance edge for amorphous oxides. As far as the authors know, this is the only work devoted to the study of the electronic structure of anodic niobia.

S2.3. Niobia IV

In ref. [35] the NbO_2 energy gap between the valence and conduction bands is 0.86 eV. In ref. [36] for epitaxial NbO_2 (110) films, 20 nm thick, were grown by pulsed laser deposition on Al_2O_3 (0001) substrates, an indirect band gap of 0.57 eV for NbO_2. Spectroscopic ellipsometry performed on a 36.8 nm thick epitaxial film of NbO_2 shows that the lowest direct gap occurs at 1.3 eV. An indirect gap may exist below 1 eV. A theoretical calculation of the dielectric function including matrix element effects shows that absorption on set and peak placement are consistent with an indirect band gap of 0.95 eV [37]. In ref. [38], the value of the NbO_2 films band gap has already been slightly corrected and was determined to be at least 1.0 eV using a combination of XPS and IPS in conjunction with hybrid density functional calculations of the density of states. The stoichiometric NbO_2 film showed an indirect band gap of 0.7 eV and direct gap of 1.24 eV [36]. The optical gaps of NbO_2 samples are 0.34, and 0.36 eV for 54 and 57 nm films [39]. Extremely doubtful, taking into account all the previously cited information, is the message that the band gap determination for NbO_2 films is less reliable due to light scattering, but a value of 3.5 eV was estimated [31], unless we assume that there is kind of direct transition. Comparing these data with the results given in ref. [40], then in Figure 3 from ref. [40], according to the results of spectroscopic ellipsometry, there are two absorption bands, and one of the transitions (obviously, a straight line) corresponds approximately to energies, even higher than 4.5 eV. An energy of 0.76±0.1 eV at room temperature was with absorbance spectra for indirect band gap determined, which is in good agreement with the spectroscopic ellipsometry data. It turns out that the absorbance band corresponding to the indirect transition in pure niobium dioxide does not fall into the wavelength range studied or is at its very beginning. The ratio of oxygen and metallic niobium in thin films Nb_2O_5 and NbO_2 deposited by plasma-enhanced atomic layer deposition is 80:20 to 40:60, and the values of the forbidden width of the optical band gap decreased from 3.91 to 2.19 eV, respectively [41]. In the case NF, PF and CF, perhaps, one can talk about an even higher proportion of niobium dioxide in the mixture of oxides in a continuous oxide layer under the columns of Nb_2O_5 and even the presence of other

sub-oxides with niobium in an even lower oxidation state (NbO). In the Nb–O system there exist numerous metastable oxides NbO_x with $0<x<1$ and $2.0<x<2.5$ as well as a multitude of Nb_2O_5 polymorphic modifications [42,43].

As can be seen, most of the analyzed results relate to oxides characterized by sufficient purity and the absence of impurities. The variety of properties is mainly due to the crystalline modification, the degree of crystallinity and deviations in the stoichiometry of the films. As for the anodic oxides of valve metals, they are X-ray amorphous [13,43–46], and are characterized not only by short-range order, but also by the presence of impurities embedded from the electrolyte [43,46–48], moreover, in some cases their content can be extremely high [49]. There is no consensus regarding the localization of impurities. Duplex [50–54], and triplex [54,55] structures of PA cell-walls are considered, but in any model of impurity distribution, the volume of anodic oxide located at the interface between adjacent oxide cells is practically free from contamination. In addition, during the formation of the described films, mutual contamination of the oxides additionally occurs. For example, in ref. [43,46], it was unambiguously shown that the film surface is mainly composed of niobium pentoxide mixed with a small portion of alumina (niobia nanocolumns contain aluminum ions). It is logical to assume that in anodic alumina, the pore walls in contact with niobia nanocolumns growing in them may contain niobium ions dissolved in it. Niobia themselves are not strictly stoichiometric, and in the nanocolumns, and even more so in the sublayer of niobium dioxide, there is an oxygen concentration gradient [43,46]. All this testifies to the fact that the systems under study are characterized by a high defective structure, which will cause an even more dramatic smearing of the band edges and the appearance of numerous electronic states in the band gap than occurs in conventional defective or amorphous materials. Features of the structure and composition of anodic oxides of valve metals, which are a consequence of the method of their formation, on the one hand, and a significant scatter of the above data and significant differences in most of the materials described in the literature from the systems studied, on the other hand, make it difficult to make a final and unambiguous decision on the assignment absorbance bands.

S3. Selecting Refractive Index of FDTD Simulation

When selecting the parameters of the model, the reference data from ref. [56] and the scarce literature data on niobia V and niobia IV were taken into account. In ref. [25] the film refractive index varied between 2.09 and 2.22 at the wavelength of 550 nm depending on the annealing temperature – with an increase in the annealing temperature, the refractive index also increases. It is also indicated in ref. [27] that the refractive index of Nb_2O_5 films has the maximum value of $n=2.37$, which is almost equal to the reported value for niobia single crystals. In ref. [57] Nb_2O_5 prepared by plasma enhanced chemical vapor deposition films exhibited a refractive index of 2.26 at 550 nm. In ref. [33] it was shown that the Nb_2O_5 refractive indices $n=2.4$ for 559 nm turned out to be highly correlated to the stress values. In ref. [58], the dependences of the refractive index and extinction coefficient were also investigated and it was shown that the refractive index values of the Nb_2O_5 thin films at 550 nm increased from 2.14 (2.18) to 2.34 (2.44) when the O_2 ratio was 10% (20%) and the refractive index values also increased with the increase of the deposition temperature. The refractive index at the same wavelength increased as the deposition temperature was increased from room temperature to 200 °C and slightly increased as the deposition temperature was further increased. The refractive index at a photon energy of 2 eV (620 nm) is approximately 2.21 for Nb_2O_5 films [31] and also states that the refractive index at a photon energy of 2 eV (620 nm) is approximately 2.38 for NbO_2 films. Reference [40] provides graphical dependences of the refractive index and extinction coefficient for NbO_2.

S4. Calculation of Native Film Refractive Index

Initial data and calculation results are presented in Table S1.

Table S1. Initial data and calculation results of the native film effective refractive index.

Direct measured data from morphology			
Native film total thickness, nm	$1.408{\cdot}10^{3}$	Empty pore diameter, nm	$2.000{\cdot}10$
PA thickness with empty pores, nm	$9.680{\cdot}10^{2}$	Column diameters, nm	$6.500{\cdot}10$
Niobia V filled PA thickness, nm	$3.250{\cdot}10^{2}$	Interpore distance, nm	$1.250{\cdot}10^{2}$
Thickness of continuous niobia layer, nm	$1.150{\cdot}10^{2}$		
Literature data			
Niobia V refractive index	2.10	Air refractive index	1.00
Alumina refractive index	1.58	Niobia IV refractive index	2.41
Calculated results			
Filling factor (porosity) of empty alumina	$2.322{\cdot}10^{-2}$	Filling factor of Niobia V filled PA	$2.45{\cdot}10^{-1}$
Empty PA effective refractive index	1.56	PA effective refractive index filled with Niobia V	1.72
Native film effective refractive index (electric field is along the direction of the structure stratification)	1.63	Native film effective refractive index (electric field is orthogonal to the direction of the structure stratification)	1.68

The upper layer with respect to the incident beam is PA with a thickness of 968 nm with pores filled with air, 20 nm in diameter and an interpore distance of 125 nm, the second (middle) layer is also PA 325 nm thick, but its pores are embedded with columns of Nb_2O_5 with a diameter 65 nm. The bottom layer is 115 nm thick continuous niobium dioxide. As a result, native film is a laminar structure, i.e., a structure formed by alternating layers with differing refractive indices, which turns out to be the simplest case of a composite medium [59], the two upper layers (empty PA and filled PA) of which are themselves nanocomposites, the effective refractive index of each of which is determined refractive indices of the components.

Thus, the calculation of the effective refractive index of this laminar structure falls into three stages. The first two should be calculations of the effective refractive indices of two nanocomposite layers based on anodic alumina, and then the effective refractive index of the entire system should be calculated.

To calculate the refractive index of composite materials, various models are used, based on certain assumptions and having certain limitations. In ref. [59], the optical properties of nanostructured semiconductors and insulators are considered based on the so-called effective-medium model. The basic idea is that the ensemble of nanoclusters can be considered a certain new medium with an effective dielectric permittivity.

To calculate the effective refractive indices of both layers, the Maxwell-Garnett model [60,61] was chosen. Firstly, this model is widely used to calculate the effective refractive index of inhomogeneous media of various kinds [62–66], and secondly, the Maxwell-Garnett model is considered valid when one of the materials forms a matrix and the other enters it as isolated inclusions whose volume fraction is not large (the so-called matrix media) [59].

The porosity or filling factor of a hexagonal structure (Equation (S2)) and the effective refractive index n_{eff} of both upper inhomogeneous layers (Equations (S3) and (S4)) is calculated according to [63–65] and for anodic alumina with pores filled with air, it was

2.32%. Filling factor of the anodic oxide with niobia columns was 24.5%. At the same time, the authors conditionally considered the first two layers to be isotropic.

$$f = \frac{\pi}{2\sqrt{3}}\left(\frac{d}{a}\right)^2 \quad \text{(S2)}$$

where d is the pore (or pore fillers) diameter and a is the interpore distance, nm.

$$\varepsilon = n^2 \quad \text{(S3)}$$

where ε is the dielectric coefficient and n is refractive index of material.

$$\varepsilon_{eff} = \varepsilon_{mat}\left(\frac{\varepsilon_f + 2\varepsilon_{mat} + 2f\left(\varepsilon_f - \varepsilon_{mat}\right)}{\varepsilon_f + 2\varepsilon_{mat} - f\left(\varepsilon_f - \varepsilon_{mat}\right)}\right) \quad \text{(S4)}$$

where ε_f and ε_{mat} are the dielectric coefficients of filler materials and matrix (alumina) respectively and f is the filling factor of filler.

In addition to the morphological parameters, the calculation required the optical characteristics of the materials that make up the film. According to [64], the refractive index of high purity alumina is 1.65. In ref. [19] thin alumina films formed by the spray pyrolysis technique have a refractive index of 1.66. In ref. [22] the Al_2O_3 films were deposited on glass, quartz, and silicon substrates by spray pyrolysis and by electron beam evaporation. The refractive index of evaporated Al_2O_3 film is 1.71 and 1.60 at 300 and 500 nm, respectively and that of sprayed film is 1.58 at 500 nm. In ref. [65] for all calculations the refractive index of the pure alumina was taken to be 1.7, but this value seems somewhat overestimated. For the calculations, the refractive index of aluminum oxide was chosen equal to 1.58. Based on the previously considered literature data on the refractive indices of niobia Nb_2O_5 and NbO_2, the refractive indices of 2.10 and 2.41, respectively, were chosen for the calculations. Then, after substitution, the result for anodic alumina with unfilled pores was 1.56, and for a layer with pores filled with Nb_2O_5 was 1.72.

Guided by work [59], and taking into account Equation (S3), using Microsoft Excel, the authors calculated the effective refractive index n_{eff} of the NF. To calculate the effective refractive index of laminar structures, the work provides two formulas for the cases if the electric field is orthogonal to the direction of the structure stratification and electric field is along the direction of the structure stratification (Equations (S5) and (S6) respectively). For the first case, the calculated refractive index was 1.68, and for the second, 1.63.

$$\varepsilon_{eff} = f_1\varepsilon_1 + f_2\varepsilon_2 + f_3\varepsilon_3 \quad \text{(S5)}$$

$$\frac{1}{\varepsilon_{eff}} = \frac{f_1}{\varepsilon_1} + \frac{f_2}{\varepsilon_3} + \frac{f_3}{\varepsilon_3} \quad \text{(S6)}$$

where $f_1 \ldots f_3$ are the filling factors for each layer.

Taking into account the laminarity of the layers, the filling factor f_i of i-th layer can be calculated according to Equation (S7)

$$f_i = \frac{H_i}{H} \quad \text{(S7)}$$

where H_i is the thickness of i-th layer, H is the total thickness of laminar structure.

References

1. Stanford, A.L.; Tanner, J.M. Early Quantum Physics. In *Physics for Students of Science and Engineering*; Elsevier: Amsterdam, The Netherlands, 1985; ISBN 978-0-12-663380-1, pp. 691–716, doi.org/10.1016/B978-0-12-663382-5.X5001-7.
2. Urbach, F. The Long-Wavelength Edge of Photographic Sensitivity and of the Electronic Absorption of Solids. *Phys. Rev.* **1953**, *92*, 1324–1324, doi:10.1103/PhysRev.92.1324.
3. Hwang, C.J. Properties of Spontaneous and Stimulated Emission in GaAs Junction Lasers. I. Densities of States in the Active

Regions. *Phys. Rev. B* **1970**, *2*, 4117–4125, doi:10.1103/PhysRevB.2.4117.
4. Kurik, M.V. Urbach rule. *Phys. Status Solidi* **1971**, *8*, 9–45, doi:10.1002/pssa.2210080102.
5. Dow, J.D.; Redfield, D. Toward a Unified Theory of Urbach's Rule and Exponential Absorption Edges. *Phys. Rev. B* **1972**, *5*, 594–610, doi:10.1103/PhysRevB.5.594.
6. Roberts, M.; Dunstan, D.J. A theory of band-gap fluctuations in amorphous semiconductors. *J. Phys. C Solid State Phys.* **1985**, *18*, 5429–5433, doi:10.1088/0022-3719/18/28/012.
7. John, S.; Soukoulis, C.; Cohen, M.H.; Economou, E.N. Theory of Electron Band Tails and the Urbach Optical-Absorption Edge. *Phys. Rev. Lett.* **1986**, *57*, 1777–1780, doi:10.1103/PhysRevLett.57.1777.
8. Sa-Yakanit, V.; Glyde, H.R. Urbach Tails and Disorder. *Comments Condens. Matter Phys.* **1987**, *13*, 35–48.
9. Bonalde, I.; Medina, E.; Rodríguez, M.; Wasim, S.M.; Marín, G.; Rincón, C.; Rincón, A.; Torres, C. Urbach tail, disorder, and localized modes in ternary semiconductors. *Phys. Rev. B* **2004**, *69*, 195201, doi:10.1103/PhysRevB.69.195201.
10. Ikhmayies, S.J.; Ahmad-Bitar, R.N. A study of the optical bandgap energy and Urbach tail of spray-deposited CdS:In thin films. *J. Mater. Res. Technol.* **2013**, *2*, 221–227, doi:10.1016/j.jmrt.2013.02.012.
11. Piccardo, M.; Li, C.-K.; Wu, Y.-R.; Speck, J.S.; Bonef, B.; Farrell, R.M.; Filoche, M.; Martinelli, L.; Peretti, J.; Weisbuch, C. Localization landscape theory of disorder in semiconductors. II. Urbach tails of disordered quantum well layers. *Phys. Rev. B* **2017**, *95*, 144205, doi:10.1103/PhysRevB.95.144205.
12. Malinenko, V.P.; Spirin, O.V. Excitation spectra of amorhous tantalum and niobium oxides. *Russ. Acad. Sci. Trans. Kola Sci. Centre. Chem. Mater. Ser. 2.* **2018**, *9*, 681–684, doi:10.25702/KSC.2307-5252.2018.9.1.681-684.
13. Reddy, P.R.; Ajith, K.M.; Udayashankar, N.K. Optical and mechanical studies on free standing amorphous anodic porous alumina formed in oxalic and sulphuric acid. *Appl. Phys. A* **2018**, *124*, 765, doi:10.1007/s00339-018-2163-7.
14. Perevalov, T.V.; Gritsenko, V.A. Application and electronic structure of high-permittivity dielectrics. *Uspekhi Fiz. Nauk* **2010**, *180*, 587, doi:10.3367/UFNr.0180.201006b.0587.
15. Schultze, J.W.; Lohrengel, M.M. Stability, reactivity and breakdown of passive films. Problems of recent and future research. *Electrochim. Acta* **2000**, *45*, 2499–2513, doi:10.1016/S0013-4686(00)00347-9.
16. Konyushenko, M.A.; Filatova, E.O.; Konashuk, A.S.; Nelyubov, A. V.; Shulakov, A.S. Experimental determination of the top of the valence band in amorphous Al_2O_3 and γ-Al_2O_3. *Tech. Phys. Lett.* **2015**, *41*, 922–925, doi:10.1134/S1063785015100077.
17. Toyoda, S.; Shinohara, T.; Kumigashira, H.; Oshima, M.; Kato, Y. Significant increase in conduction band discontinuity due to solid phase epitaxy of Al_2O_3 gate insulator films on GaN semiconductor. *Appl. Phys. Lett.* **2012**, *101*, 231607, doi:10.1063/1.4769818.
18. Afanas'ev, V.V.; Houssa, M.; Stesmans, A.; Heyns, M.M. Band alignments in metal–oxide–silicon structures with atomic-layer deposited Al_2O_3 and ZrO_2. *J. Appl. Phys.* **2002**, *91*, 3079–3084, doi:10.1063/1.1436299.
19. Aguilar-Frutis, M.; Garcia, M.; Falcony, C. Optical and electrical properties of aluminum oxide films deposited by spray pyrolysis. *Appl. Phys. Lett.* **1998**, *72*, 1700–1702, doi:10.1063/1.121156.
20. Filatova, E.O.; Konashuk, A.S. Interpretation of the Changing the Band Gap of Al_2O_3 Depending on Its Crystalline Form: Connection with Different Local Symmetries. *J. Phys. Chem. C* **2015**, *119*, 20755–20761, doi:10.1021/acs.jpcc.5b06843.
21. Canulescu, S.; Rechendorff, K.; Borca, C.N.; Jones, N.C.; Bordo, K.; Schou, J.; Pleth Nielsen, L.; Hoffmann, S. V.; Ambat, R. Band gap structure modification of amorphous anodic Al oxide film by Ti-alloying. *Appl. Phys. Lett.* **2014**, *104*, 121910, doi:10.1063/1.4866901.
22. Shamala, K.S.; Murthy, L.C.S.; Narasimha Rao, K. Studies on optical and dielectric properties of Al_2O_3 thin films prepared by electron beam evaporation and spray pyrolysis method. *Mater. Sci. Eng. B* **2004**, *106*, 269–274, doi:10.1016/j.mseb.2003.09.036.
23. Zaghdoudi, W.; Gaidi, M.; Chtourou, R. Microstructural and Optical Properties of Porous Alumina Elaborated on Glass Substrate. *J. Mater. Eng. Perform.* **2013**, *22*, 869–874, doi:10.1007/s11665-012-0322-0.
24. Emeka, N.C.; Imoisili, P.E.; Jen, T.-C. Preparation and Characterization of NbxOy Thin Films: A Review. *Coatings* **2020**, *10*, 1246, doi:10.3390/coatings10121246.
25. Rani, R.A.; Zoolfakar, A.S.; O'Mullane, A.P.; Austin, M.W.; Kalantar-Zadeh, K. Thin films and nanostructures of niobium pentoxide: Fundamental properties, synthesis methods and applications. *J. Mater. Chem. A* **2014**, *2*, 15683–15703, doi:10.1039/c4ta02561j.
26. Agarwal, G.; Reddy, G.B. Study of surface morphology and optical properties of Nb_2O_5 thin films with annealing. *J. Mater. Sci. Mater. Electron.* **2005**, *16*, 21–24, doi:10.1007/s10854-005-4953-x.
27. Venkataraj, S.; Drese, R.; Liesch, C.; Kappertz, O.; Jayavel, R.; Wuttig, M. Temperature stability of sputtered niobium–oxide films. *J. Appl. Phys.* **2002**, *91*, 4863–4871, doi:10.1063/1.1458052.
28. Raba, A.M.; Bautista-Ruíz, J.; Joya, M.R. Synthesis and Structural Properties of Niobium Pentoxide Powders: A Comparative Study of the Growth Process. *Mater. Res.* **2016**, *19*, 1381–1387, doi:10.1590/1980-5373-mr-2015-0733.
29. Dash, J.K.; Chen, L.; Topka, M.R.; Dinolfo, P.H.; Zhang, L.H.; Kisslinger, K.; Lu, T.-M.; Wang, G.-C. A simple growth method for Nb_2O_5 films and their optical properties. *RSC Adv.* **2015**, *5*, 36129–36139, doi:10.1039/C5RA05074J.
30. Lenzmann, F.; Shklover, V.; Brooks, K.; Grätzel, M. Mesoporous Nb_2O_5 films: influence of degree of crystallinity on properties. *J. Sol-Gel Sci. Technol.* **2000**, *19*, 175–180, doi:10.1023/A:1008767717625.
31. Hossain, N.; Günes, O.; Zhang, C.; Koughia, C.; Li, Y.; Wen, S.-J.; Wong, R.; Kasap, S.; Yang, Q. Structural and physical properties of NbO_2 and Nb_2O_5 thin films prepared by magnetron sputtering. *J. Mater. Sci. Mater. Electron.* **2019**, *30*, 9822–9835, doi:10.1007/s10854-019-01319-8.

32. Santos, A. Nanoporous anodic alumina photonic crystals: fundamentals, developments and perspectives. *J. Mater. Chem. C* **2017**, *5*, 5581–5599, doi:10.1039/C6TC05555A.
33. Hunsche, B.; Vergöhl, M.; Neuhäuser, H.; Klose, F.; Szyszka, B.; Matthée, T. Effect of deposition parameters on optical and mechanical properties of MF- and DC-sputtered Nb_2O_5 films. *Thin Solid Films* **2001**, *392*, 184–190, doi:10.1016/S0040-6090(01)01025-2.
34. Jia, Y.; Zhong, M.; Yang, F.; Liang, C.; Ren, H.; Hu, B.; Liu, Q.; Zhao, H.; Zhang, Y.; Zhao, Y. Theoretical and Experimental Study on Exciton Properties of TT-, T-, and H-Nb_2O_5. *J. Phys. Chem. C* **2020**, *124*, 15066–15075, doi:10.1021/acs.jpcc.0c04202.
35. Roberson, J.A.; Rapp, R.A. Electrical properties of NbO and NbO_2. *J. Phys. Chem. Solids* **1969**, *30*, 1119–1124, doi:10.1016/0022-3697(69)90368-0.
36. Joshi, T.; Senty, T.R.; Borisov, P.; Bristow, A.D.; Lederman, D. Preparation, characterization, and electrical properties of epitaxial NbO_2 thin film lateral devices. *J. Phys. D: Appl. Phys.* **2015,** *48*, 335308, doi:10.1088/0022-3727/48/33/335308.
37. O'Hara, A.; Nunley, T.N.; Posadas, A.B.; Zollner, S.; Demkov, A.A. Electronic and optical properties of NbO_2. *J. Appl. Phys.* **2014**, *116*, 213705, doi:10.1063/1.4903067.
38. Posadas, A.B.; O'Hara, A.; Rangan, S.; Bartynski, R.A.; Demkov, A.A. Band gap of epitaxial in-plane-dimerized single-phase NbO_2 films. *Appl. Phys. Lett.* **2014**, *104*, 092901, doi:10.1063/1.4867085.
39. Wong, F.J.; Hong, N.; Ramanathan, S. Orbital splitting and optical conductivity of the insulating state of NbO_2. *Phys. Rev. B* **2014**, *90*, 115135, doi:10.1103/PhysRevB.90.115135.
40. Stoever, J.; Boschker, J.E.; Bin Anooz, S.; Schmidbauer, M.; Petrik, P.; Schwarzkopf, J.; Albrecht, M.; Irmscher, K. Approaching the high intrinsic electrical resistivity of NbO_2 in epitaxially grown films. *Appl. Phys. Lett.* **2020**, *116*, 182103, doi:10.1063/5.0005523.
41. Lee, S.-H.; Kwon, J.-D.; Ahn, J.-H.; Park, J.-S. Compositional and electrical modulation of niobium oxide thin films deposited by plasma-enhanced atomic layer deposition. *Ceram. Int.* **2017**, *43*, 6580–6584, doi:10.1016/j.ceramint.2017.02.089.
42. Pligovka, A.; Yunin, P.; Hoha, A.; Korolyov, S.; Gorokh, G.; Skorokhodov, E. Morphology and Structure of Defected Niobium Oxide Nonuniform Arrays Formed by Anodizing Bilayer Al/Nb Systems. *Tech. Phys.* **2020**, *65*, 1771–1776, doi:10.1134/S1063784220110213.
43. Mozalev, A.; Vázquez, R.M.; Bittencourt, C.; Cossement, D.; Gispert-Guirado, F.; Llobet, E.; Habazaki, H. Formation–structure–properties of niobium-oxide nanocolumn arrays via self-organized anodization of sputter-deposited aluminum-on-niobium layers. *J. Mater. Chem. C* **2014**, *2*, 4847, doi:10.1039/c4tc00349g.
44. Poznyak, A.; Pligovka, A.; Laryn, T.; Salerno, M. Porous Alumina Films Fabricated by Reduced Temperature Sulfuric Acid Anodizing: Morphology, Composition and Volumetric Growth. *Materials.* **2021**, *14*, 767, doi:10.3390/ma14040767.
45. Alcalá, G.; Skeldon, P.; Thompson, G.E.; Mann, A.B.; Habazaki, H.; Shimizu, K. Mechanical properties of amorphous anodic alumina and tantala films using nanoindentation. *Nanotechnology* **2002**, *13*, 302, doi:10.1088/0957-4484/13/4/302.
46. Mozalev, A.; Vázquez, R.M.; Calavia, R.; Bittencourt, C.; Gispert-Guirado, F.; Llobet, E.; Hubálek, J.; Habazaki, H. Development of multipurpose metal-oxide films with nanostructured tailored morphologies and tunable properties via anodic processing of al/NB metal layers. In Proceedings of the 4th International Conference — NANOCON´12, Brno, Czech Republic, 23–25 Oct. 2012; pp. 33–38.
47. Tajima, S.; Baba, N.; Shimura, F.M. Einfluss von Anionen und Inhibitoren auf die primären wachstumsvorgänge der anodischen Oxidschichten auf Aluminium. *Electrochim. Acta* **1967**, *12*, 955-IN2, doi:10.1016/0013-4686(67)80095-1.
48. Wood, G.C.; Skeldon, P.; Thompson, G.E.; Shimizu, K. A Model for the Incorporation of Electrolyte Species into Anodic Alumina. *J. Electrochem. Soc.* **1996**, *143*, 74–83, doi:10.1149/1.1836389.
49. Golovataya, S. V.; Zubarevich, O.I.; Knornschild, G.; Muravitskaya, E. V.; Poznyak, A.A. Highly doped nanostrcturized template of anodic alumina. In Proceedings of the 2008 18th International Crimean Conference – Microwave & Telecommunication Technology; IEEE, 2008; pp. 581–583.
50. Lee, W.; Park, S.J. Porous anodic aluminum oxide: Anodization and templated synthesis of functional nanostructures. *Chem. Rev.* **2014**. *114*, 7487–7556, doi:10.1021/cr500002z.
51. Thompson, G.. Porous anodic alumina: fabrication, characterization and applications. *Thin Solid Films* **1997**, *297*, 192–201, doi:10.1016/S0040-6090(96)09440-0.
52. Law, C.S.; Lim, S.Y.; Abell, A.D.; Voelcker, N.H.; Santos, A. Nanoporous Anodic Alumina Photonic Crystals for Optical Chemo- and Biosensing: Fundamentals, Advances, and Perspectives. *Nanomaterials* **2018**, *8*, 788, doi:10.3390/nano8100788.
53. Brzózka, A.; Brudzisz, A.; Rajska, D.; Bogusz, J.; Palowska, R.; Wójcikiewicz, D.; Sulka, G.D. Recent trends in synthesis of nanoporous anodic aluminum oxides. In *Nanostructured Anodic Metal Oxides*; Elsevier: Amsterdam, The Netherlands, 2020; ISBN 9780128167069, pp. 35–88, doi:10.1016/B978-0-12-816706-9.00002-9.
54. Sulka, G.D. Highly Ordered Anodic Porous Alumina Formation by Self-Organized Anodizing. In *Nanostructured Materials in Electrochemistry*; Wiley-VCH: Weinheim, Germany, 2008; ISBN 9783527621507, pp. 1-116, doi:10.1002/9783527621507.ch1.
55. Thompson, G.E.; Wood, G.C. Anodic Films on Aluminium. In *Treatise on Materials Science and Technology*; Elsevier: Amsterdam, The Netherlands, 1983; ISBN 9780126336702, pp. 205–329, doi:10.1016/B978-0-12-633670-2.50010-3.
56. Gao, L.; Lemarchand, F.; Lequime, M. Exploitation of multiple incidences spectrometric measurements for thin film reverse engineering. *Opt. Express* **2012**, *20*, 15734, doi:10.1364/OE.20.015734.
57. Szymanowski, H.; Zabeida, O.; Klemberg-Sapieha, J.E.; Martinu, L. Optical properties and microstructure of plasma deposited Ta_2O_5 and Nb_2O_5 films. *J. Vac. Sci. Technol. A Vacuum, Surfaces, Film.* **2005**, *23*, 241–247, doi:10.1116/1.1851544.

58. Chen, K.-N.; Hsu, C.-M.; Liu, J.; Liou, Y.-C.; Yang, C.-F. Investigation of Antireflection Nb_2O_5 Thin Films by the Sputtering Method under Different Deposition Parameters. *Micromachines* **2016**, *7*, 151, doi:10.3390/mi7090151.
59. Golovan, L.A.; Timoshenko, V.Y.; Kashkarov, P.K. Optical properties of porous-system-based nanocomposites. *Physics-Uspekhi* **2007**, *50*, 595, doi:10.1070/PU2007v050n06ABEH006257.
60. Maxwell Garnett, J.C. XII. Colours in metal glasses and in metallic films. *Philos. Trans. R. Soc. London. Ser. A, Contain. Pap. a Math. or Phys. Character* **1904**, *203*, 385–420, doi:10.1098/rsta.1904.0024.
61. Maxwell Garnett, J.C. VII. Colours in metal glasses, in metallic films, and in metallic solutions.—II. *Philos. Trans. R. Soc. London. Ser. A, Contain. Pap. a Math. or Phys. Character* **1906**, *205*, 237–288, doi:10.1098/rsta.1906.0007.
62. Skryabin, I.L.; Radchik, A. V.; Moses, P.; Smith, G.B. The consistent application of Maxwell–Garnett effective medium theory to anisotropic composites. *Appl. Phys. Lett.* **1997**, *70*, 2221–2223, doi:10.1063/1.118821.
63. Zhang, D.; Zhang, H.; He, Y. In-situ thickness measurement of porous alumina by atomic force microscopy and the reflectance wavelength measurement from 400–1000 nm. *Microsc. Res. Tech.* **2006**, *69*, 267–270, doi:10.1002/jemt.20311.
64. Xu, Q.; Yang, Y.; Gu, J.; Li, Z.; Sun, H. Influence of Al substrate on the optical properties of porous anodic alumina films. *Mater. Lett.* **2012**, *74*, 137–139, doi:10.1016/j.matlet.2012.01.076.
65. Choudhari, K.S.S.; Sangeeth, K.; Kulkarni, S.D.; Chidangil, S. Optical Interferometric Properties of Porous Anodic Alumina Nanostructures. *Mater. Today Proc.* **2016**, *3*, 2443–2449, doi:10.1016/j.matpr.2016.04.160.
66. Millán, C.; Santonja, C.; Domingo, M.; Luna, R.; Satorre, M.Á. An experimental test for effective medium approximations (EMAs). *Astron. Astrophys.* **2019**, *628*, A63, doi:10.1051/0004-6361/201935153.
